# Effect of Obesity and Other Risk Factors on Hypertension among Women of Reproductive Age in Ghana: An Instrumental Variable Probit Model

**DOI:** 10.3390/ijerph16234699

**Published:** 2019-11-26

**Authors:** Abayomi Samuel Oyekale

**Affiliations:** Department of Agricultural Economics and Extension, North-West University, Mafikeng Campus, Mmabatho 2735, South Africa; asoyekale@gmail.com

**Keywords:** hypertension, cardiovascular diseases, women, reproductive age, Ghana

## Abstract

*Background*: The growing incidence of mortality as a result cardiovascular diseases (CVDs) is a major public health concern in several developing countries. In Ghana, unhealthy food consumption pattern and sedentary lifestyle are promoting overweight and obesity, with significant consequences on the incidence of CVDs. Specifically, hypertension morbidity is now a public health concern among Ghanaian health policy makers. This paper analysed the effect of body mass index (BMI)/arm circumference and other associated factors on hypertension risk among women of reproductive ages in Ghana. *Methods*: The data were collected as Demographic and Health Survey (DHS) in 2014. This paper analysed the subset of the data that were collected from eligible women 15–49 years of age. The total sample was 9396, while 9367 gave consents to have their blood pressure measured. Data were analysed with instrumental probit regression model with consideration of potential endogeneity of BMI and arm circumference. *Results*: The results showed that 25% of the women were either overweight or obese, while 13.28% were hypertensive. Women from the Greater Accra (18.15%), Ashanti (15.53%) and Volta (15.02%) regions had the highest incidences of hypertension. BMI and arm circumferences were truly endogenous and positively associated with the probability of being hypertensive. Other factors that influenced hypertension were age of women, region of residence, urban/rural residence, being pregnant, access to medical insurance, currently working, consumption of broth cubes, processed can meats, salted meat and fruits. *Conclusion*: It was concluded that hypertension risk was positively associated with being overweight, obesity, age and consumption of salted meat.It was inter aliaemphasized that engagement in healthy eating with less consumption of salted meats, and more consumption of fruits would assist in controlling hypertension among Ghanaian women.

## 1. Background

The spate of non-communicable diseases (NCDs) in many developing countries is now of serious concern to health policy makers. Moreover, of significant concern is increase in the number of people that are currently suffering from overweight and obesity, which are health conditions where the individual’s body mass index (BMI) is in the range of 25 and 30 kg/m^2^ and ≥30 kg/m^2^, respectively [1,2]. These conditions have been linked to excessively raised blood pressure that requires some medical attentions. According to Adler et al. [3], a blood pressure of ≥140 mm Hg for systolic or ≥90 mm Hg for diastolic is clinically considered high, thereby requiring some medical assistances. Individuals with normal BMI (18.5–24.9 kg/m^2^) are medically susceptible to very low risk of NCDs, while overweigh and obese individuals live at a very high risk of hypertension, cardiovascular diseases (CVDs), musculoskeletal disorders, diabetes and some form of cancerous infections [4,5,6].

Globally, it had been estimated that since 1975, prevalence of obesity has almost tripled, with 39% and 13% of the adult population (18 years and above) being overweight and obese respectively in 2016 [7]. It was also noted that in 2016, 17.9 million deaths—which represented 31% of global estimated deaths—were due to CVDs [8]. In 2015, developing countries accounted for 82% of untimely deaths resulting from NCDs, 37% of which was caused by CVDs [8]. Therefore, the battles against chronic diseases like hypertension, diabetes and their associated renal and cardiovascular complications are daily attaining status of alarming proportions. More importantly, hypertension currently portrays the ruthlessness of any chronic disease and spontaneously denies the conventional notion of its direct association with affluences of economically developed countries. Obviously, therefore, the premonition of its insignificance as a health problem in economically developing countries some decades ago has rapidly faded away, with Africa now accounting for one of its highest health burdens.

Moreover, hypertension now ranks as the major cause of untimely death in low- and middle-income countries, where about 80% of all cardiovascular mortality had been estimated to occur [9]. Available statistics have also shown that hypertension constitutes significant health burdens in low income countries, especially those in Africa, where the double-edged sword of highly infectious and chronic diseases poses significant threats to socioeconomic development [10].

However, it is ironical that in many developing countries, serious attention that the growing burdens of obesity and cardiovascular diseases deserve is yet to be given, or at most being addressed with some provocatively political leap services [11]. In Ghana, obesity and hypertension are health problems of significant public health concern [12,13]. Although prevalence of overweight and obesity in Ghana varies based on the segment of population that had been studied; a systematic review by Ofori-Asenso et al. [12] reveals that national prevalence of obesity was 17.1% among Ghanaian adults. Over the years, moreover, new cases of outpatient hypertension increased by more than ten times from 49,087 in 1988 to 505,180 in 2007 [14]. Some other studies have noted some progressive increases in the prevalence of NCDs in Ghana [15,16,17]. Available statistics indicate that among Ghanaian adults who were 45 years and above, hypertension took the second place as the cause of outpatient morbidity, while NCDs accounted for 22.2% of deaths at one of the foremost Teaching Hospital in 2013 [18]. 

This study seeks to analyse the effect of obesity and other factors on hypertension risk among women of reproductive age in Ghana. Besides the fact that obesity influences hypertension, it is also important to note that the unexplainable variables that promote hypertension may as well influence obesity thereby reinvigorating serious endogeneity problem. This study contributes to existing knowledge by addressing this suspected endogeneity using instrumental variable approach. In addition, data on arm circumferences were used as proxy for obesity, in order to evaluate its impact on hypertension for the larger segment of the respondents since data on BMI were missing for many of the respondents. Using a suitable econometric approach, we tested the null hypothesis that obesity does not significantly increase the risk of hypertension among Ghanaian women.

## 2. Methods

### 2.1. Study Area

This study was conducted in Ghana, which is one of the Anglophone countries in West Africa. The country is administratively divided into ten regions—Greater Accra, Ashanti, Western, Central, Volta, Eastern, Brong Ahafo, Northern, Upper East, and Upper West—which are further sub-administered within some 216 districts [19]. The Ghana’s human development indices increased from 0.588 in 2016 to 0.592 in 2017 with life expectancy respectively increasing from 62.7 years to 63 years. The human development indicator for 2017 ranked Ghana as 140^th^ in the world, while her gender inequality index of 0.538 was ranked as 131^st^ [20].

### 2.2. Data and Sampling Procedures

Data for this study were obtained from the website of the Demographic and Health Survey (DHS) with comprehensive description of data collection procedures highlighted in Ghana Statistical Service et al. [21]. Ethical approval for the study was granted by “the Ghana Health Service Ethical Review Committee and the Institutional Review Board of ICF International” [21]. The data were collected in 2014 by Ghana Statistical Service using the updated sampling framework of the Ghana’s 2010 Population and Housing Census [19]. The data were collected using two-stage sampling design, taking cognizance of the need to ensure national representativeness with critical understanding of country’s urban-rural and regional population composition. At the first stage, 216 and 211 clusters were selected in urban and rural areas respectively [21]. Systematic sampling was used to randomly select the sampled households after comprehensively listing the households in each of the selected EAs between January and March 2014. From each of the selected EAs, 30 households were selected. This implies selection of a total of 12,831 households, of which 12,010 were occupied and 11,835 completed the survey. In addition, this paper analysed the data that were collected from eligible women 15–49 years of age. In the rural and urban areas, there were 9656 eligible women, of which 9396 completed the survey. This study made use of the data from 9367 women that gave consent to havingtheir blood pressure measured. 

### 2.3. Estimated Model

Modelling obesity and hypertension risk factors requires some econometric procedures with due consideration of their potential endogeneity. The conventional Probit model only produces unbiased estimators when endogeneity is not a problem within the specified model. In this study, two stage Probit model was used in order to detect and correct the likelihood of obesity being endogenous within the specified hypertension risk model. The ideal procedure is to specify the main structural model as:

(1) $$HP_{i}=\alpha +\beta_{i}X_{i}+\gamma BMI_{i}+e_{i}$$

(2) $$BMI_{i}=\delta +\partial_{i}X_{i}+\mu WT_{i}+\rho SE_{i}+\;v_{i}$$

In Equation (1), *HP_i_* is a dummy variable coded as 1 for hypertensive respondents and 0 otherwise. Hypertensive individuals are those whohad been medically confirmed to have raised blood pressures that require regular intake of some medications and those for which the average values of their last two blood pressure measurements for systolic or diastolic were ≥140 mm Hg or ≥90 mm Hg, respectively. The Xs is a vector of explanatory variables such as woman’s age, residence in rural areas (yes 1, 0 otherwise), wife to the household’s head (yes 1, 0 otherwise), daughter to the household’s head (yes 1, 0 otherwise), gender of head (female 1, 0 otherwise), currently pregnant (yes 1, 0 otherwise), smoking no tobacco (yes 1, 0 otherwise), holds health insurance (yes 1, 0 otherwise), number of unions, currently working (yes 1, 0 otherwise), processed can fish/meat added to food (yes 1, 0 otherwise), salted dried meat added to food (yes 1, 0 otherwise), broth cubes added to food (yes 1, 0 otherwise), days ate fruits in past 7 days, days ate vegetables in past 7 days and number of trips in the past one month. 

BMI is the body mass index of women. It should be noted that this variable was missing for the majority of the women because their weights and heights were not recorded. In order to proceed with the analysis with the full sample of those whose blood pressures were measured, a proxy for BMI was sought. From the dataset, the arm circumferences of women were measured. This was taken as a proxy for BMI in the analysis for the full sample. This can be justified from several studies that had found arm circumference to be highly correlated with obesity, thereby making it a good measure of BMI [22,23]. Equations (1) and (2) can therefore be specified as Equations (3) and (4), respectively:

(3) $$HP_{i}=\omega +\varphi_{i}X_{i}+\tau AC_{i}+k_{i}$$

(4) $$BMI_{i}=\theta +\vartheta_{i}X_{i}+\varepsilon WT_{i}+\epsilon SE_{i}+\;s_{i}$$

where *AC* denotes the values of women’s upper arm circumferences.

In this study, we suspected that BMI or its proxy would be endogenous. Endogeneity problem arises if the error component in Equation (1) is correlated with the error term of Equation (2). This can be the case given that some key omitted variables (like stress), which are drivers of hypertension, can as well drive obesity [24].

### 2.4. Selection of Instruments

Instrument selection is a major procedure in estimating instrumental variable Probit regression. Beside the econometric properties which the instruments must possess, there is the need for ensuring some theoretical justification and relevance. A quick review of the literature provides the needed directions in selecting instrumental variable within the orbits of the proposed study. Following some previous studies [25,26,27], Dang [28] analysed the effect of body weight on hypertension in Vietnam using the offspring’s body weight as instrumental variable. It was found that body weight increased the likelihood of being hypertensive, although significant variations existed within various age intervals. A recent study by Lee et al. [29] considered endogeneity of obesity in explaining hypertension in Korea using the Mendelian randomization design. Using individual’s genetic risk score to instrument body mass index, it was found that a causal relationship existed between BMI and hypertension. 

In this study, the BMI of parents and siblings of the women were not measured. The other anthropometric data were collected from children 0–60 months of age. However, this information was only available for about 25% of the total women. In order to safeguard losing a lot of the respondents due to missing data, efforts were put at finding another instrumental variable. The closest variable to physical activity in the data is time to source of drinking water (variable *WT* in Equation (2)). This was considered as an instrument for BMI given that obesity is not completely a genetic issue. Specifically, it had been noted that increase in obesity among people with constant genetic pool suggests the increasing contributions of some environmental factors [30]. In many instances, modernization brings along some costly alteration to human lifestyles with increased consumption of fast food and physical inactivity [31,32] Therefore, recent increase in the prevalence of obesity is largely attributed to physical inactivity and changes in feeding lifestyle with more intake of fat, carbohydrate and high energy drinks [33].

The second instrument was the years of education of the husband (*SE* in Equation (2)). There are different ways through which education could influence obesity. Although some studies have highlighted the role of education in profitable engagement in the labour markets [34], other studies have confirmed the role of education in promoting other non–monetary returns such as good health and well-being [35,36,37]. Although the direction of association between education and obesity could be positive or negative, many of the studies that were conducted in low income countries showed positive association [38,39], while those in developed countries showed negative association [40,41]. Given the selected instruments, the Instrumental Variable Probit equations are specified as:

(5) $$HP_{i}=\xi +\varpi_{i}X_{i}+\pi BMI_{i}+\sigma v_{i}+m_{i}$$

(6) $$HP_{i}=\omega +\varphi_{i}X_{i}+\pi^{*}AC_{i}+\sigma^{*}v_{i}+n_{i}$$

However, if the parameter of *v_i_* is not statistically significant, this implies that the model could be estimated using the standard Probit regression. The analyses were carried out with STATA 13 software, which provides Wald test of exogeneity (athrho = 0). Statistical significance of this parameter implies that endogeneity is present and that the null hypothesis should be rejected.

## 3. Results

### 3.1. Incidences of Hypertension and Obesity

Table 1 shows the distribution of women’s BMI. It reveals that one out of every four women was overweight. Moreover, four out of every ten women would either be overweight or obese. Table 2 shows the blood pressure readings across some of their demographic characteristics. It also reveals the classifications of the respondents’ Body Mass Index (BMI). It reveals that 10.40% of the women had high blood pressure readings with systolic or diastolic being respectively higher or equal to 140 mm Hg and 90 mm Hg. Moreover, 66.03% of the women had optimal blood pressure with systolic and diastolic being respectively lower than 120 mm Hg and 80 mm Hg. In addition, 15.28% and 8.29% had normal and high normal blood pressure readings, respectively. However, 7.04%, 2.08% and 1.29% of the women had mild, moderate and severe hypertension, respectively. The results further show that young women (<35 years) accounted for the highest proportions of the women in the optimal and normal blood pressure groups. These results also show that women that were 35 years and above accounted for the highest proportions in the hypertensive groups.

Across the regions, optimal blood pressure readings were mostly recorded in the Northern (79.55%), Upper East (76.82%) and Upper West (75.45%). Furthermore, 13.53% of the women in Greater Accra had high blood pressure readings. This was followed by 12.85% in the Ashanti region and 11.75% in the Volta region. Table 2 further shows that across the sectors of the Ghanaian economy, 12.47% of urban women had high blood pressure readings as compared to 8.00% for rural women. The results in Table 2 also show that except for women with <20 cm arm circumference, the proportions that were with high blood pressure readings increased as the arm circumferences increased. Moreover, as the women’s BMI increased, the proportions with high blood pressure also increased.

Table 3 presents the comprehensive incidences of hypertension across the women’s demographic characteristics. These results were computed with inclusion of those women who were confirmed to be hypertensive by either nurses or doctors in the category of those suffering from hypertension, even if their blood pressure readings were in the normal range as a result of being currently engaged in some medical treatments. It reveals that the proportion of all the women with hypertensive health condition was 13.28%. These proportions increased as the age groups increased. Specifically, among women who were 40–45 and 45–50 years of age, hypertension incidences were 24.98% and 39.44%, respectively.

Table 3 also shows that across the regions, percentages of hypertensive women were highest in the Greater Accra (18.15%), Ashanti (15.53%) and Volta (15.02%). However, hypertension incidences were lowest in the Upper West (6.05%), Upper East (7.54%) and Northern regions (8.03%). In addition, the incidence of hypertension was higher in urban areas (16.34%), compared to rural areas (9.73%). The table further shows the distribution of hypertension incidences across the different range of arm circumferences and body mass index. Specifically, 62.22% and 39.01% of the women with arm circumference of ≥45 cm and 40–45 cm were respectively hypertensive. Similarly, the incidences of hypertension increased as the women’s body mass indices increased. Precisely, 31.54% and 15.31% of obese and overweight women were respectively hypertensive. 

### 3.2. Obesity and Demographic Determinants of Hypertension

The results in Table 4 were estimated to determine the effect of obesity on hypertension. The explanatory variables were first subjected to multicollinearity test, which showed that there was no serious correlation among the explanatory variables. This was confirmed by low values (1.63) of the computed variance inflation factor (VIF). The results were estimated for the full sample where arm circumference was used as a proxy for the missing BMI variable. In addition, sub-sample analyses were carried out with women with body mass index values. In all the results that have been presented in Table 4, the model properly fitted the data. This is explained by the significance of the Wald Chi Square statistics (*p* < 0.05). In the results for the full sample, analyses were carried out with the dependent variables being those women with high blood pressure or those with medically confirmed hypertension coded as one and zero otherwise. In the other results in columns four and five, only those with confirmed hypertension were coded as one, others were coded as zero. However, the arm circumference was used as a proxy for BMI while education of the women’s husbands and time to water source were used as instrumental variables. The results for the Wald’s test of exogeneity showed that arm circumference and BMI were truly endogenous. This is confirmed from statistical significance of the computed statistics (*p* < 0.05). The results show that as arm circumference increases, probability of being hypertensive significantly increased (*p* < 0.05). In the results obtained for sub-samples, as BMI and arm circumferences increased, probability of being hypertensive also significantly increased (*p* < 0.05).

Across all the results, the parameters of women’s age did not show statistical significance except in the second sub-sample model where its parameter is with positive sign and statistically significant (*p* < 0.01). From the regional perspectives, the parameters of Central and Upper West regions are with negative sign and statistically significant (*p* < 0.05) in the first and second full sample models. These results indicate that compared to women from the Western region, women who were resident in the Central and Upper West regions had significantly lower probability of being hypertensive. In addition, compared to those from Western region, women who were resident in Northern region had significantly higher probability of being medically confirmed to be hypertensive.

The parameters of being currently pregnant are statistically significant (*p* < 0.05) in all the estimated models except for the one that was estimated in the second full sample model. These parameters indicate that compared to women who were not pregnant, women who were currently pregnant had lower probability of being hypertensive. Similarly, in all the models, the estimated parameters for currently working variable are with negative sign and statistically significant (*p* < 0.05). These results imply that compared to those women who were not working, women in the labour market had lower probability of being hypertensive. 

Some variables were included in the model in order to reflect nutrition and cooking lifestyles. Specifically, the parameters that were estimated for cooking with broth cubes variable all have negative sign across all the models and statistically significant (*p* < 0.05) except for that in the second full sample model. These results generally show that compared to women who did not use broth cubes for cooking, women who were cooking with broth cubes had lower probability of being hypertensive. Similar results were obtained for the variable “cook with processed can meats”. The estimated parameters for the first and second full models are with negative sign and statistically significant (*p* < 0.05). These results also indicate that women who were cooking with processed can meats had significantly lower probability of being hypertensive. However, the parameters of the variable, “cook with salted dried meat” show statistical significance (*p* < 0.05) in the full sample models and with positive sign. These imply that women who were cooking with salted dried meat had higher probability of being hypertensive. The parameters of the number of days ate fruits are with negative sign and statistically significant (*p* < 0.05) in the full sample models. These results show that as the number of days that women ate fruits increase, their probability of being hypertensive decreased. 

## 4. Discussion

The results show that 10.28% of the women had high blood pressure readings. However, when those with medically diagnosed hypertension were included, hypertension incidence increased to 13.28%. This result depicts a relatively high incidence of hypertension when compared with 8% that was estimated for women of similar age groups (20–44 years) in the United States of America (USA) [42]. Chen and Chauhan [43] also estimated hypertension among women of reproductive age in USA as 8.5%. The estimated prevalence is however lower than 30% that was estimated for rural women in Haiti [44]. In Zambia, it was reported that among women of child-bearing age (18–45 years), incidences of hypertension were 18.6% and 6.7% in Lusaka and Chibombo, respectively [45]. Another study in Brazil estimated hypertension incidences as 15.1% in 2008 and 14.7% in 2015 [46].

The results indicated that more than half of the women were with normal BMI. This can be compared to results from Punjab, where 46.3% of the women had normal BMI [47]. With 15.48% of the women being obese, Ghanaian women have shown low body weights in comparison with what had been reported in some previous studies. Specifically, among African American, 30.7% were found to be severely obese [48,49]. In a study by Amugsi et al. [50], it was found that between 1995 and 2014, obesity doubled in Benin, Kenya, Niger, Rwanda, Ivory Coast and Uganda, but tripled in Zambia, Burkina Faso, Mali, Ghana, Malawi and Tanzania. Egyptian women had the highest levels of overweight (44%) and obesity (39%) while women from Ethiopia and Madagascar had the lowest incidences of obesity and overweight. It was noted that being obese or overweight may be less stigmatized among African American, thereby inducing less pressure on the need to lose weight [51,52]. 

BMI and arm circumferences were found to be positively related with hypertension. These results are in line with the findings from some previous studies [47,53,54,55,56,57,58,59]. There are therefore compelling evidences of positive association between obesity and hypertension as well as other CVDs [60]. Evidence aboundsonthe health benefits of losing weight. A study had shown reduction in systolic blood pressure by as much as 5.4% and 40% improvement in insulin resistance due to average of 8.9% reduction in weight by some therapeutic changes in lifestyles [61]. Some other authors have highlighted the health benefits that can result from 5% to 10% loss in body weights [62,63]. In another study, significant and positive correlation had been found between obesity and some cardiometabolic risk factors depending on the cut-off point for obesity [64].

The results further emphasized the role of age in explaining hypertension incidences. It was observed that as the women’s age groups increased, the proportions with hypertension also increased. Similar findings have been reported among some women in the USA [42,65]. In another study, it was noted that hypertension among aged people results from stiffness of arteries due to persistent age-related structural changes [66]. Pinto [66] also noted that there are tendencies for stiffness of the large artery as a result of some arteriosclerotic structural alterations and calcification resulting from ageing. It was further added that ageing increases peripheral vascular resistance (PVR) in small blood vessels. These two conditions are interrelated and account for increase in systolic blood pressure, although DBP increases with PVR.

We also found some regional and sectoral differences in the incidences of hypertension among Ghanaian women. Specifically, Greater Accra and Ashanti regions had the highest incidences. These results may be due to high level of urbanization in those regions. In addition, hypertension incidence among rural dwellers was lower than what obtained among urban residents. This finding is contrary to that of Wang et al. [67] who found prevalence of hypertension in rural areas of China to be higher than that in urban areas. Hypertension prevalence in urbanized society may be promoted by less engagement in physical activities, involvement in multiple jobs, devotion of less time to walking, consumption of salty junk foods and exposure to high levels of stress [67].

The results also show that being pregnant reduced the probability of suffering from hypertension among women of reproductive ages in Ghana. This variable was included to capture the influences that gestational and preeclampsia hypertension could have on the dependent variable. Another vital issue is that hypertensive disorder in women is related to the gestational period of the pregnancy. During the first trimester, there are tendencies towardsreduction in the blood pressure [68]. In a study by Singh et al. [69], hypertensive disorder was found among 17% of selected pregnant women in Nigeria, while preeclampsia was at 6%.

The results also indicate that women with health insurance had higher probability of being hypertensive in one of the full sample results. This result is in line with our expectation because women that are suffering from hypertension may have a higher likelihood of subscribing to health insurance. It had been noted that inadequate access to medical insurance is a critical constraint to accessing effective hypertension treatments [70]. It had been found that uninsured hypertensive patients were more than four times likely to have unmet medical prescriptions [71] and low treatments than their insured counterparts [72]. Similar findings have been reported in other studies [73], although Brooks et al. [74] did not find significant relationship between hypertension medical treatments and uptake of health insurance.

In addition, the women that were working had significantly lower probability of being hypertensive. This result can be explained from some perspectives. Depending on the nature of job, labour market participation can reduce inactivity and financial stress which are culprits of several CVDs [75,76]. Similarly, some jobs may be so sedentary and strenuous to the extent of inducing development of some CVDs [75,76]. In another dimension, loss of job can induce significant psychological disturbances, which can facilitate development of some CVDs. In a study in the USA, it was found that job loss and unemployment episodes were significantly associated with some acute cardiovascular events [77]. Similarly, it had been reported that involuntary loss of job more than twice increased the risk of developing myocardial infarction and stroke [78]. Our results are in alignment with some previous studies that found negative association between labour market engagements or number of working hours and hypertension [79,80].

The results also highlighted the importance of nutrition in controlling blood pressure. Specifically, contrary to expectations, women that were cooking with broth cubes and processed can meat had lower probability of being hypertensive. However, in line with expectations, cooking with salted meat increased the probability of being hypertensive. The sodium contents of cooking products like broth cubes and processed can meats hasraised some dietary concerns among healthcare professionals [81,82,83,84,85,86]. World Health Organization [87] recommended consumption of maximum of 5 g of salt (1.5 g of sodium) per day. It is therefore imperative to note that while broth cubes and processed meats contain salt, it is excessive consumption that is detrimental to people’s health. Therefore, cooking with broth cubes and processed can meat may not automatically imply high intake of sodium, depending on the quantity that is being served and consumed per meal. 

In a study in Ghana [88], it was noted that while bouillon cubes “contain as much as 50% to 60% of salt”, and it could serve as a vital source of iodine. It was noted that results of a recent survey reveal that almost 80% of the respondents consumed bouillon in about 11 to 15 times in the week preceding the survey. This is equivalent to about 20 g of bouillon (2.8 g/day), estimated to contain 9.7 g of salt (1.4 g/day). This implies that 28% of an average adult person’s daily requirement of salt was obtained from consuming bouillon cubes.

In essence, food therapy with low sodium contents is often recommended for individuals at prehypertensive stage, while drug therapy would only be the option after food therapy has been proved ineffective. Some clinical trials have highlighted the effect of sodium intake on prevention of hypertension [81], ensuring effectiveness of BP control among those on antihypertensive medication [82,83]. In some other observational studies, reduction in the intake of sodium is associated reduction in the risk of atherosclerotic cardiovascular and congestive heart failure [84,85,86].

Vegetable and fruit consumption are often advocated as part of the components of healthy eating [89]. The parameters of the number of days that fruits were consumed within a week show statistical significance (*p* < 0.05) with negative sign in the full models. This finding is in line with expectation because intake of fruits increases the consumption of viscous soluble fibre that had been found to reduce overall SBP and DBP in many studies [89,90].

## 5. Conclusions

This paper evaluates the association between obesity and hypertension risk among women of reproductive age in Ghana. The analyses were further implemented using arm circumference as a proxy for BMI with due consideration of their endogeneity. The results have shown that BMI and arm circumference were truly endogenous and positively associated with the probability of being hypertensive. Other factors that influenced hypertension were age of women, region of residence, urban/rural residence, being pregnant, access to medical insurance, currently working, and consumption of broth cubes, processed can meats, salted meat and fruits. It can be concluded that modelling hypertension risk in absence of data on BMI can utilize arm circumference information with less loss of analytical efficiency. Moreover, hypertension risk would reduce among women if public health policy targets activities that can reduce overweight and obesity among women. Similarly, hypertension consciousness should be promoted among older women, urban residents and those in Kumasi and Greater Accra. Healthy eating with less consumption of sodium and the need to promote more consumption of fruits should be emphasized.

## Figures and Tables

**Table 1:** Distribution of women’s body mass index.

BMI	Frequency	Percent	Cumulative %
Underweight	277	5.83	5.83
Normal	2543	53.62	59.45
Overweight	1189	25.07	84.52
Obese	734	15.48	100.00

**Table 2:** Distribution of women’s blood pressure readings in Ghana.

Demographic Group	Optimal		Normal		High Normal		Mild High		Moderate High		Severely High		Total High	% High
Age groups	Frequency	%	Frequency	%	Frequency	%	Frequency	%	Frequency	%	Frequency	%		
<20	1346	82.89	201	12.40	45	2.74	31	1.93	1	0.03	0	0.01	32	1.97
20–25	1 254	78.10	203	12.62	97	6.06	47	2.95	4	0.25	0	0.02	52	3.22
25–30	1 180	73.66	222	13.87	99	6.21	74	4.65	15	0.96	10	0.65	100	6.27
30–35	879	64.27	226	16.52	115	8.41	99	7.24	31	2.24	18	1.33	148	10.80
35–40	704	54.50	241	18.67	170	13.18	119	9.19	40	3.08	18	1.39	176	13.66
40–45	502	49.00	180	17.53	140	13.61	129	12.56	39	3.80	36	3.51	204	19.87
45–50	321	37.62	159	18.62	110	12.93	160	18.74	65	7.69	38	4.40	263	30.83
Total	6 185	66.03	1 431	15.28	776	8.29	659	7.04	195	2.08	120	1.29	975	10.40
Regions														
Western	671	64.69	167	16.09	103	9.89	68	6.53	19	1.83	10	0.97	97	9.33
Central	633	67.73	147	15.73	67	7.15	57	6.09	16	1.73	15	1.58	88	9.39
Greater Accra	1 083	57.15	336	17.71	220	11.61	168	8.88	47	2.46	41	2.19	256	13.53
Volta	472	65.64	108	15.02	55	7.58	62	8.68	14	1.94	8	1.13	85	11.75
Eastern	601	69.08	118	13.61	69	7.95	49	5.66	24	2.81	8	0.89	81	9.36
Ashanti	1 134	63.55	283	15.88	138	7.72	152	8.54	48	2.70	29	1.61	229	12.85
BrongAhafo	528	68.85	119	15.52	54	7.02	49	6.37	11	1.50	6	0.74	66	8.61
Northern	625	79.55	80	10.17	38	4.87	32	4.06	8	0.99	3	0.35	42	5.41
Upper East	274	76.82	44	12.31	19	5.36	14	3.90	5	1.44	1	0.18	20	5.52
Upper West	163	75.45	29	13.41	14	6.51	7	3.42	2	0.96	1	0.26	10	4.63
Sector													0	0.00
Urban	3 128	62.09	802	15.93	479	9.51	402	7.97	134	2.67	92	1.83	628	12.47
Rural	3 057	70.61	629	14.53	297	6.86	258	5.95	61	1.40	28	0.65	346	8.00
Arm Circumference														
<20	103	59.75	31	17.75	17	9.86	15	8.94	2	1.10	4	2.59	22	12.64
20–25	622	79.46	81	10.39	47	5.98	24	3.08	8	0.99	1	0.10	33	4.17
25–30	3 023	76.31	521	13.16	220	5.55	142	3.58	40	1.01	15	0.38	197	4.97
30–35	1 888	58.41	565	17.47	326	10.07	313	9.68	82	2.53	60	1.86	454	14.06
35–40	452	47.56	180	18.92	125	13.10	117	12.29	52	5.44	26	2.70	194	20.43
40–45	86	38.68	48	21.42	30	13.30	36	16.27	10	4.46	13	5.88	59	26.61
≥45	11	24.33	6	13.21	13	28.43	12	27.47	2	3.90	1	2.65	15	34.02
BMI														
Missing	3 117	66.66	724	15.47	368	7.86	304	6.50	112	2.39	52	1.11	468	10.00
Underweight	224	81.77	21	7.64	22	7.87	5	1.76	2	0.86	0	0.11	7	2.72
Normal	1 861	74.01	315	12.54	169	6.70	127	5.05	26	1.03	17	0.67	170	6.75
Overweight	668	56.79	232	19.70	134	11.35	100	8.52	22	1.84	21	1.80	143	12.16
Obese	315	43.34	140	19.26	85	11.69	123	16.98	33	4.57	30	4.16	187	25.71
Doctor told of hypertension														
No	6 069	68.53	1 356	15.31	698	7.88	542	6.12	123	1.39	69	0.78	733	8.28
Yes	116	22.73	75	14.76	78	15.31	118	23.02	72	14.08	52	10.11	241	47.21

**Table 3:** Distribution of Hypertension Incidences among Women in Ghana.

	No Hypertension		Hypertension	
Age Groups	Frequency	%	Frequency	%
<20	1589	97.84	35	2.16
20–25	1536	95.70	69	4.30
25–30	1477	92.20	125	7.80
30–35	1169	85.45	199	14.55
35–40	1067	82.65	224	17.35
40–45	769	75.02	256	24.98
45–50	516	60.56	336	39.44
All respondents	8123	86.72	1244	13.28
Regions				
Western	926	89.21	112	10.79
Central	837	89.52	98	10.48
Greater Accra	1551	81.85	344	18.15
Volta	611	84.98	108	15.02
Eastern	753	86.45	118	13.55
Ashanti	1507	84.47	277	15.53
Brong Ahafo	683	89.05	84	10.95
Northern	722	91.97	63	8.03
Upper East	331	92.46	27	7.54
Upper West	202	93.95	13	6.05
Sector of the Economy				
Urban	4215	83.66	823	16.34
Rural	3908	90.27	421	9.73
Arm Circumference				
<20	150	87.21	22	12.79
20–25	738	94.37	44	5.63
25–30	3707	93.59	254	6.41
30–35	2666	82.46	567	17.54
35–40	709	74.55	242	25.45
40–45	136	60.99	87	39.01
≥45	17	37.78	28	62.22
Body Mass Index				
Missing	4065	86.93	611	13.07
Underweight	260	95.24	13	4.76
Normal	2305	91.61	211	8.39
Overweight	996	84.69	180	15.31
Obese	497	68.46	229	31.54

**Table 4:** Results of instrumental probit regression of effect of obesity on hypertension.

	Full Sample				Sub-Sample			
	High BP and Medically Confirmed Hypertension		Medically Confirmed Hypertension Only		High BP and Medically Confirmed Hypertension		High BP and Medically Confirmed Hypertension	
	Coefficient.	z-stat	Coefficient	Z-stat	Coefficient.	z-stat	Coefficient	Z-stat
BMI	-	-	-	-	0.1754 ***	3.54	-	-
Arm circumference	0.2134 ***	8.27	0.2388 ***	11.30	-	-	0.0013 ***	3.01
Age of the women	0.0127	1.35	0.0007	0.07	0.0223	1.56	0.0320 ***	2.93
Rural area	0.0355	0.56	0.0640	0.86	−0.0259	−0.26	−0.0132	−0.13
Regions								
Central	−0.2024 ***	−2.86	−0.3524 ***	−4.10	−0.1779	−1.60	−0.0154	−0.14
Greater Accra	−0.1789	−1.78	−0.2539 **	−2.38	−0.0390	−0.25	0.1068	0.84
Volta	−0.0705	−0.86	−0.0643	−0.67	0.0010	0.01	0.2600 **	2.29
Eastern	−0.1045	−1.43	−0.0978	−1.18	−0.0785	−0.71	0.1000	0.89
Ashanti	0.0017	0.02	−0.0615	−0.78	−0.1187	−1.09	−0.0150	−0.14
Brong Ahafo	0.0052	0.08	−0.0462	−0.58	−0.0018	−0.02	0.1502	1.25
Northern	0.1452	1.92	0.2368 ***	3.02	0.0378	0.31	0.2140	1.29
Upper East	−0.0497	−0.67	0.0219	0.27	−0.1132	−1.01	0.1432	0.95
Upper West	−0.1688 **	−2.09	−0.2142 **	−2.07	−0.1306	−1.10	0.1143	0.75
Relationship with the head								
Wife to the Head	0.0013	0.02	0.0005	0.00	0.0228	0.19	0.0583	0.44
Daughter to the Head	0.1136	1.59	0.1331	1.59	0.0926	0.86	0.0504	0.45
Head is Female	0.1102	1.55	0.0713	0.78	0.1028	0.94	0.1240	1.07
Currently Pregnant	−0.1756 **	−1.99	0.0255	0.33	−0.3018 **	−2.29	−0.5189 ***	−4.41
Does not smoke	0.0083	0.08	−0.0600	−0.58	0.3667	0.91	0.2331	0.56
Has health insurance	0.0361	0.94	0.1341 **	2.05	0.0584	1.07	0.0139	0.22
Number of Unions	0.0236	0.68	0.0480	1.05	0.0620	1.32	0.0618	1.28
Currently Working	−0.1562 ***	−3.96	−0.1614 ***	−3.45	−0.1417 **	−2.26	−0.1331 **	−1.99
Cook with broth cubes	−0.0505 **	−2.13	−0.0243	−0.96	−0.0853 **	−2.31	−0.0991 **	−2.54
Cook with Processed can meats	−0.0818 **	−2.20	−0.1063 ***	−2.84	−0.0590	−1.04	−0.0097	−0.17
Cook with Salted dried meat	0.0567 **	2.03	0.0884 ***	2.77	0.0609	1.49	0.0735	1.75
Number of days ate fruits	−0.0144 **	−2.09	−0.0257 ***	−3.55	−0.0152	−1.44	−0.0179	−1.55
Number of days ate vegetables	0.0039	0.55	0.0135	1.79	−0.0028	−0.26	−0.0056	−0.52
No. of trips in the past 12 months	−0.0044	−1.68	−0.0076 ***	−2.83	−0.0050	−1.26	−0.0039	−0.96
Constant	−7.5261 ***	−20.18	−8.1314 ***	−40.74	−7.1926 ***	−7.85	−5.5169 ***	−7.50
Athrho	−0.8511 ***	−3.61	−1.1000 ***	−3.43	−0.5942 **	−2.01	−0.4155	−1.76
Lnsigma	1.3584 ***	185.93	1.3585 ***	185.92	1.3420 ***	130.70	6.0789 ***	592.06
Rho	−0.6916		−0.8005		−0.5329		−0.3931	
Sigma	3.8900		3.8903		3.8267		436.5483	
Number of observations	9367		9367		4743		4743	
Wald chi2(26)	2905.26 ***		3120.61 ***		915.11 ***		706.08 ***	
Wald test of exogeneity chi2(1)	13.05 ***		11.79 ***		4.03 **		3.09 *	

Note: *** Statistically significant at 1% level; ** Statistically significant at 5% level; * Statistically significant at 10% level.

## Data Availability

The data for this study were obtained from the DHS website. The author was granted the permission to use the data.

## Abbreviations

BMI	Body Mass Index
CVDs	Cardiovascular Diseases
DBP	Diastolic Blood Pressure
DHS	Demographic and Health Survey
NCDs	Non-Communicable Diseases
PVR	Peripheral Vascular Resistance
SBP	Systolic Blood Pressure
USA	United States of America
VIF	Variance Inflation Factor
